# Expert Systems for Predicting the Bioavailability of Sun Filters in Cosmetic Products, Software vs. Expert Formulator: The Benzophenone-3 Case

**DOI:** 10.3390/pharmaceutics14091815

**Published:** 2022-08-29

**Authors:** Anna Baldisserotto, Erika Baldini, Sara Ravarotto, Elena Cesa, Daniela De Lucia, Elisa Durini, Silvia Vertuani, Stefano Manfredini, Bozena B. Michniak-Kohn

**Affiliations:** 1 Department of Life Sciences and Biotechnology, Section of Medicinal and Health Products, University of Ferrara, Via Fossato di Mortara 17-19, 44121 Ferrara, Italy; 2 Department of Life Sciences and Biotechnology, University of Ferrara, Via Fossato di Mortara 17-19, 44124 Ferrara, Italy; 3 DLD Scientific LLC, United State Corporation Agent, Inc., Pine Brook, NJ 07058, USA; 4 Center for Dermal Research (CDR) & Laboratory for Drug Delivery (LDD) Life Sciences Building, Rutgers—The State University of New Jersey, 145 Bevier Road, Piscataway, NJ 08854, USA

**Keywords:** Formulating for Efficacy^®^ Software, Benzophenone-3, sunscreens UV filters, expert formulator

## Abstract

There are only a limited number of molecules in a cosmetic formulation, which can passively cross the stratum corneum and be absorbed into the skin layers. However, some actives should never cross the skin in large concentrations due to their potential for side effects, for example, sunscreens. Artificial intelligence is gaining an increasing role as a predictive tool, and in this regard, we selected the Formulating for Efficacy^®^ Software to forecast the changes in bioavailability of selected topical cosmetic compounds. Using the Franz diffusion cell methodology, various oils were selected as those with low release capability, and these were compared to those suggested by the software in Benzophenone-3-containing formulations. The software was able to predict the lipophilic phases, which, if utilized in the emulsion, were stable and sometimes even more pleasant in appearance and consistency than the reference emulsions prepared by the formulator. To date, however, Formulating for Efficacy^®^ Software still has limitations as far as predicting the hydrophilic phase, as well as not being able to choose the emulsifier or the preservative system.

## 1. Introduction

The skin is a very effective but selective barrier, which evolved to keep the inside environment (water and electrolytes) in and outside one (chemicals, allergens, microbes) out.

In this context, the ingredients in a topical formulation, which in the case of cosmetics can number up to several dozen, can play a significant role in facilitating the crossing of the active compound(s) through the stratum corneum. This role is misunderstood and little investigated. Differently from what one may expect, this aspect may be particularly important if one wants to reduce the capability of some specific active molecules to cross the stratum corneum, as, for example, UV sunscreens filters [1], whose toxicity may be a problem if they are allowed to cross the stratum corneum into the deeper skin layers. The components of a formulation can favor in a significant manner the passage of substances, which alone would not cross the stratum corneum. This event should be foreseen at the design stage and not simply verified at the usage stage. The trial-and-error approach to formulation is the most practiced approach in the field, but with the increased requirements by regulatory authorities and users’ sensitivity to rumors, amplified by social networks, it is becoming a long and costly process. Artificial intelligence is gaining increasing importance in the screenings of R&D processes, thus leading to consistent saving of time and unwanted side effects. Therefore, the primary aim of this study was to assess the possibility to devise formulations able to reduce the possibility of potential irritating ingredients (i.e., organic sunscreens) to penetrate the skin, and secondly, to challenge an expert system’s time-saving performance to predict the ability of a given emollient to “keep actives on the skin” and not “to deliver” an ingredient to the skin. This kind of “prediction” is usually performed by an expert formulator in the field that understands the behavior of sunscreens with regard to a chosen formulation based on their own experience and on a trial-and-error approach. With the increasing use of artificial intelligence [2] and continued interest in sunscreens and in their safety [3], we were intrigued by the Formulating for Efficacy^®^ Software (FFE Software).

As recently well summarized in a review by Steven Abbott [4], an integrated approach to skin delivery should take into account at least five principles. (a) The first is the maximum solubility in a single compatible solvent, not overcome by any solvent combination. (b) The actives and solvents will be distributed in different parts of the finished product, which can be in line with the principle predicted by the calculations. (c) The diffusion through skin can be designed, taking into account the concentration gradient coefficients, which, in turn, depend on the hindering of the molecule and the number of solvents at each level in the skin. (d) A pharmaceutical ingredient contest is much different than a cosmetic one. The latter is much more complicated due to the number of use restrictions posed by hundreds of reasons from the regulatory authorities, the properties not liked by the market, the costs, the texture, the smell and so on. (e) Last but not least, the necessity to perform this work at the physiological level with “non therapeutic actives” and in low concentrations brings the formulator in competition with “finite doses” rather than “infinite” doses, as in the pharmaceutical field, and with the use of synergic ingredients, which brings the formulation scenario in competition with multiple ingredients that may influence the five principles. As stated above, in the past years, the attempts to overcome the solubility-based concept in the cosmetic field, to rationalize the right concentration and the right formulation for efficacy, have been attempted with the use of polarity, introducing the relative polarity index (RPI) [5] and, in particular, by introducing the Hansen solubility parameters in practice model (HSPiP) and the Formulating for Efficacy^®^ Software [4,6].

The reader is reminded of the above references to better understand the basis of the Formulating for Efficacy^®^ Software approach. So far, this approach is more known in the industry field rather than in academia and, in particular, in the cosmetics area, both for the specific needs of the formulators in the field (complex mixtures and very short time to market) and for the dissemination work of the late Professor Johan Wiechers in the cosmetics literature [5], articles at conferences and especially in his teaching courses.

Taking all of this together, this approach is very interesting but needs to be validated by challenging it in representative systems in relevant, finite dose experiments.

In this preliminary study, we challenged the ability of the software to provide, through lipophilic phase changes, a correct prediction in terms of the release between the optimized formulations TO SKIN and TO ACTIVE, whose ingredients were chosen by an expert formulator, the latter relying on years of experience and knowledge of ingredients rather than on calculations.

### 1.1. Mathematical Models

In order to clarify the transport mechanisms and barrier functions of the skin, the skin can be considered as a membrane, or a set of membranes, for the study of which, various mathematical principles are applied.

Transport through the stratum corneum is a passive diffusion process governed by Fick’s law of diffusion, which correlates with direct proportionality the rate of absorption–flux to the concentration of the substance dissolved in the vehicle and to the surface of application, while it is inversely proportional to the thickness of the stratum corneum [7]. Fick’s law takes into account various parameters (such as the permeability coefficient, absorption area, thickness of the membrane crossed), which, although they are all calculable, due the complexity of the corneal barrier, they make Fick’s law only a guide. In fact, many biological and physico-chemical factors that influence percutaneous absorption are not considered, such as age, sex, race, atomic position or skin integrity [8].

### 1.2. Formulating for Efficacy (FFE)

#### 1.2.1. Theory behind FFE: HSP Parameters

The Hansen solubility parameters (HSP) were developed by Charles M. Hansen in 1967 with the aim of finding a valid mathematical model that could predict the ability of one material to solubilize in another.

The basic idea of the model is “like dissolves like”. This was translated into specific and measurable chemical parameters (in MPa):

D = describes the van der Waals forces (energy from dispersion forces); 

P = describes the polarity of the compound (energy from dipolar intermolecular force);

H = describes the hydrogen bonds (energy from hydrogen bonds) [9].

**Figure 1:**
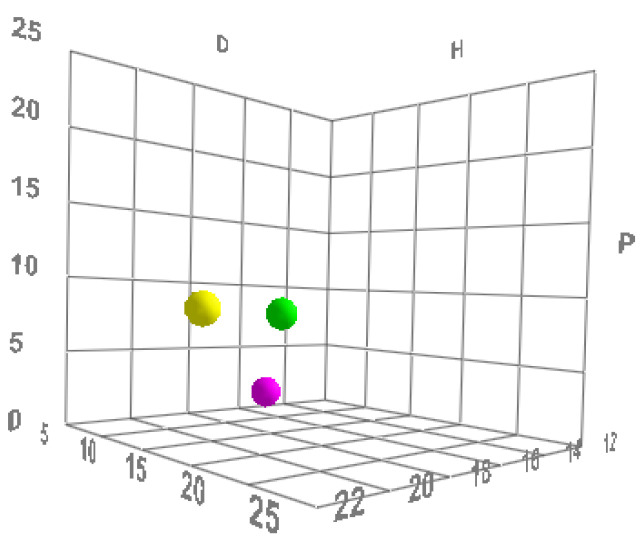
Illustrative representation of three different elements (formulation, active and skin) that are placed, according to their HSPs, in Hansen’s space. Image of a graph created by Formulating for Efficacy^®^ Software, using the three-dimensional Hansen representation. D = energy from dispersion forces; P = energy from dipolar intermolecular force; H = energy from hydrogen bonds. Reprinted and adapted with permissions from [9,10].

All molecules (including polymers) possess these three parameters and can therefore be described mathematically and graphically according to a three-dimensional graphical representation model, called the “Hansen space”, in which substances are represented as spheres, and the center of the sphere is identified using Hansen’s three parameters [4,9] (Figure 1).

The dimensions of the individual spheres are variable. The radius is the component that determines the “field of activity” of a substance, while the volume of the sphere of an active shows the limits of the space within which solvents can be graphically placed.

**Figure 2:**
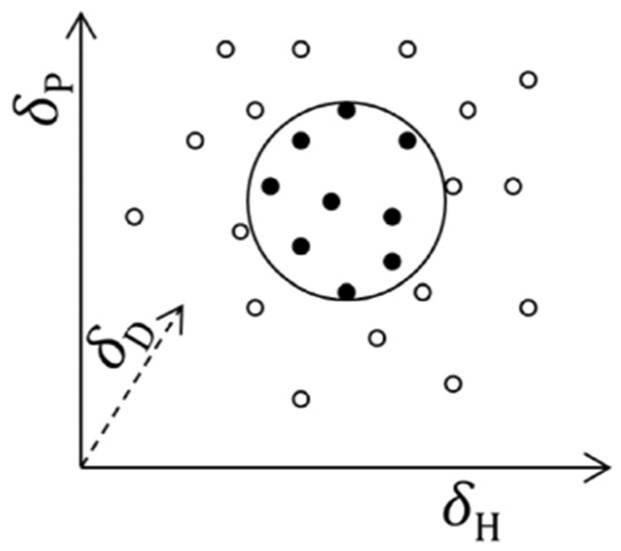
Graphic representation of an active (large sphere), a set of “good” solvents (black spheres within the active sphere) and a set of “bad” solvents (white spheres) in Hansen’s space. Reprinted and adapted with permissions from [9,10].

The greater the degree of overlap between the two spheres (for example, an active and a solvent), the greater the ability of a substance to solubilize the active (Figure 2) [9,11].

**Figure 3:**
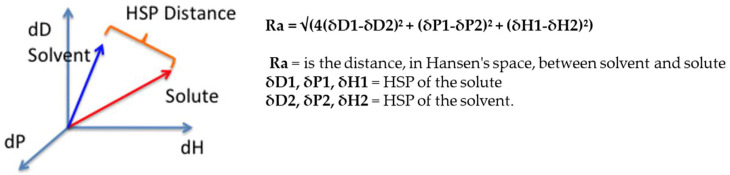
Equation and graphical representation of Ra (HSP distance) between a solute and a solvent in Hansen’s space. Reprinted and adapted with permissions from [9,10].

To ensure good solubilization of the active, the solvent must come as close as possible to the sphere representing the solute. The two components are therefore separated by a certain distance, which is measurable (Figure 3).

To best solubilize an active, it is useful to consider mixtures of substances as solvents, since the individual HSPs of each component are added and averaged; it is therefore possible to create a new solvent with HSPs (total), which are very close to those of the active, thus shortening the HSP distance.

All of this led to a very important conclusion, namely that two solvents not appropriate for a solute, when combined correctly, can create an effective one [9].

This theory was applied by Hansen himself to the skin. For years, the ability of a molecule to cross the SC was considered through the water/octanol partition coefficient (log P). The importance of this coefficient lies in its ability to indicate the level of hydrophilicity or hydrophobia of a chemical.

The log P can be calculated mathematically from the two experimentally determined solute concentrations. The values assumed by log P are typically negative for substances with a high hydrophilic character, while they are positive and increasing as the hydrophobic character increases.

As stated in Fick’s law, there is a direct proportionality between log P and the permeability coefficient. Hydrophobic substances, i.e., with a high log P, pass the SC well. However, according to Hansen’s observation, the system is not sufficiently comprehensive [10]; there are, in fact, hydrophilic substances that can pass well through the skin barrier. This is also explained anatomically. The stratum corneum is mainly composed of keratinocytes, which, consisting of proteins and water, are an important hydrophilic compartment and allow substances to pass through intracellularly. Fick’s law would seem to describe mainly the intercellular passage of substances (through extracellular lipids), so Hansen proposed a mathematical model, which took more account of the molar volume (MVol) than of the log P [10].

From these considerations and from Hansen’s work to represent the skin in a three-dimensional space [12], experimentally obtaining its HSPs, in 2004, J. Wiechers and S. Abbott created the Formulating for Efficacy^®^ Software project.

In this program, the skin is represented in Hansen’s space as a solute and is the main reference to be considered.

#### 1.2.2. Description of the Formulating for Efficacy^®^ Software

Formulating for Efficacy^®^ is a software program that proposes a systematic approach for the formulation of topical products. The aim of the program is to produce, following several parameters, an optimal lipophilic phase to improve the skin permeability of a lipophilic active.

“The Formulating for Efficacy^®^ Software calculates the optimal composition of the oil phase of a formulation for a given lipophilic active at a given concentration ” [13].

The software will initially ask the operator to select the type of formulation desired, specifying the percentage of the lipophilic phase. Next, the operator is asked to select an active with which they intend to develop the project. The actives must be lipophilic, and it is necessary to specify whether they are solubilized in a vehicle, or whether they are in a solid state. At this point, one can proceed by querying the program, thus requesting the qualitative and quantitative composition of the best lipophilic phase to obtain the desired skin passage of the active.

### 1.3. Evaluation of UV-Filter Release

In this context, it was therefore decided to devise a new study capable of completing the range profile of the software and assessing its formulation and predictive potential, using the Franz cells to compare the release data from both known formulations and formulations proposed and/or modified by the software.

The active ingredient chosen for this study was Benzophenone-3 (BP-3), a chemical sun filter, the use of which in commercial formulations is limited due to its various unfavorable characteristics, the most negative of which is undoubtedly its ability to penetrate the skin and become available for systemic absorption [1].

In this regard, the literature data report the ability to modulate the release of the sun filters through formulation strategies [1,11,14,15,16,17].

Taking this into account, the software approach for the prediction of a TO ACTIVE formula seemed as a good way to create a lipophilic phase, which is particularly affinitive to the molecule, and to retain as much as possible of the active in the formulation, thus reducing skin absorption.

Therefore, the main purpose of this work was to verify whether:

- the software was effectively capable of modulating the percutaneous passage of active substances;
- the absorption data provided by the software were reliable;
- the software could help reduce the release of BP-3 (taken, for example, from a molecule with potential adverse effects) from cosmetic formulations in a controlled manner, modifying the lipophilic phase qualitatively and quantitatively.

## 2. Materials and Methods

### 2.1. Materials

Benzophenone-3 (ACEF, Fiorenzuola, Italy), Paraffinum liquidum (Vaseline oil Bfr 0020 Ep—ACEF, Fiorenzuola, Italy), Dicaprylyl carbonate (Cetiol CC—BASF, EUROTRADING S.p.A., Veggiano, Padua, Italy), Cocogliceride (Myritol 331—BASF, EUROTRADING S.p.A., Veggiano, Padua, Italy), Caprilic-Capric triglyceride (Myritol 318—BASF, EUROTRADING S.p.A., Veggiano, Padua, Italy), Dicaprylyl ether (Cetiol OE—BASF, EUROTRADING S.p.A., Veggiano, Padua, Italy), C12–C15 Alkyl benzoate (Finsolv TN-O—innospec performance chemicals italia s.r.l, Castiglione delle Stiviere, Mantua, Italy), Tribehenin PEG-20 esters (Emulium 22—Gattefossé-Italia S.r.l., Milan, Italy), Cetearyl alcohol (Tego Alkanol 16/18—ACEF, Fiorenzuola, Italy), Squalane (Squalane Vegetale—ACEF, Fiorenzuola, Italy), Dibutyl adipate (Cetiol B—BASF, EUROTRADING S.p.A., Veggiano, Padua, Italy), Glycerin (Glycerin 30 BE°—Zetalab, Padua, Italy), Disodium EDTA (Edeta BD—BASF, EUROTRADING S.p.A., Veggiano, Padua, Italy), Carbomer (Carbopol Ultrez 10—Lubrizol-Italia S.r.l., Milan, Italy), preservative system 1,2-Hexanediol, 1,2-Octanediol and Tropolone (Symdiol 68 T—Symrise S.r.l. Milan, Italy), NaOH solution 10% (Sodium Hydroxide pellets-MERCK-SIGMA ALDRICH, Milan, Italy), Phosphate Buffer pH 7.4 (Phosphate Buffer Solutions 1.0 M—MERCK-SIGMA ALDRICH, Milan, Italy), Polysorbate-20 (TEGO SML 20—Evonik Italia S.r.l., Pandino, Cremona, Italy), Synthetic regenerated cellulose membrane (Medicell Membranes Ltd., Greenwich London, UK), EtOH (Ethanol 95.0%—MERCK-SIGMA ALDRICH, Milan, Italy), demineralized water.

### 2.2. Calculations

In this work, we used the Formulating for Efficacy^®^ Software (ACT-Solutions, University of Toledo, Health Science Campus, College of Pharmacy and Pharmaceutical Sciences, Toledo, OH, USA) to devise a standard formulation. This approach uses an innovative software assisted approach, based on Hansen’s solubility parameters (HSP), which optimizes the percentages of the components of the oil phase of an emulsion in relation to the chemical structure and function of the selected active ingredient.

**Figure 4:**
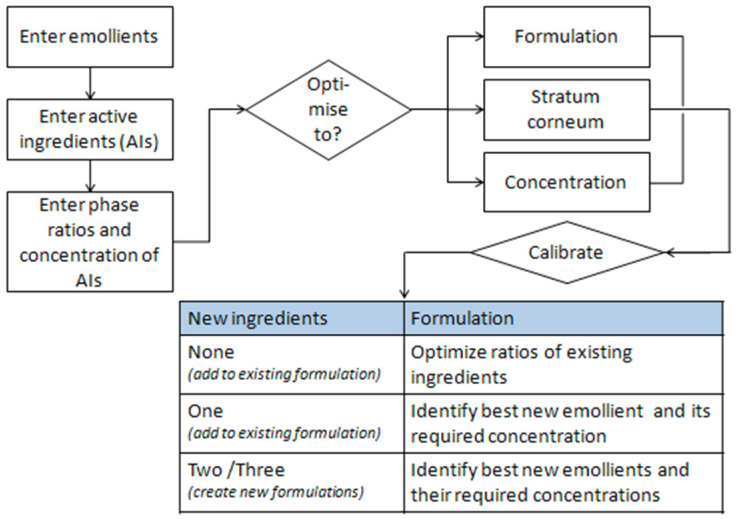
Diagram explaining the functionality of Formulating for Efficacy^®^ Software. Reprinted and adapted with permissions from [9,10].

The software helps identify the right emollients that simultaneously dissolve the active ingredients and guide them into the skin. A diagram of the software is shown below (Figure 4).

### 2.3. Preparation of the Oils

An amount of 5% (*w/w*) of benzophenone-3 was solubilized in each selected oil (Table 1). All formulations were cold prepared, with the exception of O5, which was slightly heated to obtain a complete solubilization of the active.

In the first solvent (O1, paraffinum liquidum), the active was not solubilized; therefore, the formulation was not analyzed in Franz’s cell. In oils O2–O6, BP-3 was perfectly solubilized at the chosen concentration.

### 2.4. Preparation of Emulsions

All emulsions contained 5% BP-3 (*w/w*) and were prepared according to the English method. The composition of the hydrophilic phase, the emulsifier and the preservative system were the same for each preparation.

They were solubilized in water, glycerin and EDTA after being weighed appropriately. The solution was heated to 70 °C and then stirred using a turboemulsifier (Silverson L5M-A, Crami Group S.r.l., Pero, Milan, Italy), after which Carbomer was slowly added and the system left to stir.

After weighing the ingredients of the lipophilic phase (different for each formulation prepared) and the active ingredient, the mixture was heated to 70 °C, slowly added to the hydrophilic phase and emulsified using the turboemulsifier. The preparation was cooled in a water bath at room temperature while stirring. To complete the emulsions, the preservative system was added, and finally, the formulations were adjusted to pH 6 (approximately) with 10% NaOH.

### 2.5. Composition of the Emulsions

Table 2 shows the ingredients present in the different formulations: Emulsion 1 (E1), containing O5 designed by the expert formulator; Emulsion 2 (E2), containing O6 designed by the expert formulator; Emulsion 3 (E3), software-derived TO SKIN emulsion, derived from E1; Emulsion 4 (E4), TO ACTIVE emulsion developed by the software, derived from E1; Emulsion 5 (E5), software-derived TO SKIN emulsion, derived from E2; Emulsion 6 (E6), TO ACTIVE emulsion developed by the software, derived from E2.

### 2.6. Franz Cells

Vertical glass Franz cells with a receiving compartment capacity of 4.3 mL and an absorption area of 0.6 cm^2^ were used. Regenerated synthetic cellulose membranes were chosen to conduct the permeability experiments and were hydrated in the receiving medium (16 h before use) at a temperature of 4 °C. The synthetic membrane employed in the Franz diffusion cells experiments in this study was a Cuprophan Regenerated cellulose from Medicell (London, UK), membrane thickness (11.5 μm ± 0.5 μm), molecular-weight cut off range (10,000 Da). The effective diffusion area of the Franz cells was 0.6 cm^2^, and the receptor volume was 4.3 mL.

The receiving medium chosen was a solution consisting of phosphate buffer pH 7.4, Tween-20 (2%) and ethanol (30%).

The cells, after being appropriately loaded with the recipient medium and assembled, were immersed in a thermostatically controlled bath at 32 ± 1 °C for 30 min, prior to loading the formulation. An amount of 1 mL of the formulation (approximately equivalent to 1 g) was loaded into each donor compartment (time zero). During the total 6 h of the experiment, 0.5 mL of the recipient medium (later replaced by new solution at room temperature) was withdrawn at the following times: 30 min (t1), 1 h (t2), 2 h (t3), 3 h (t4), 4 h (t5), 5 h (t6), 6 h (t7).

Each sample (oils O2–O6 and Emulsions E1–E6) was diluted with 2 mL of the solution (phosphate buffer, Tween-20, ethanol) before being analyzed by the UV-VIS methodology (SHIMADZU UV-2600, Shimadzu Italia S.r.l., Milan, Italy). The absorbance values were obtained considering the absorption peak of Benzophenone-3, at max = 324 nm. A calibration line and a release curve were constructed for each analysis. The calibration lines were created by taking 2.5 mL of a stock solution (0.01% solution of BP-3 in the receiving medium) and diluting it (with the receiving medium) at a ratio of 1:2, 9 times, progressively. The 9 different samples were analyzed using the UV-VIS methodology.

### 2.7. Data and Statistical Analysis

The cumulative active release of BP-3 and the quantity permeated per unit area were plotted against time. The results were also expressed as the percent release rate. All the results were reported as mean ± SD (n = 4). The statistical analysis of the data was performed by using one-way Anova and Student’s *t*-test, and *p*-values < 0.05 were considered significant.

## 3. Results

As a first step, the Formulating for Efficacy^®^ Software was asked, after setting up the necessary data for the creation of O/W emulsions and the data relating to the active ingredient (BP-3), to formulate a lipophilic phase with total freedom, being able to choose from various ingredients already present in the database. However, the lipophilic phases proposed by the software, both TO SKIN and TO ACTIVE, proved to be unstable and not formulable.

It was therefore decided to change the approach and start from simpler systems, i.e., from oleolites, thus taking away the software’s freedom to choose the ingredients.

Six oils were selected to begin the study, and the release data predicted by the software were compared with those obtained in vitro with Franz cells. Despite the discrepant results between the software predictions and the in vitro data, it was decided to continue the investigation by developing more complex formulations (O/W emulsions).

The program was then asked to rework the lipophilic phases of emulsions designed by an experienced formulator, containing the previously selected oils. It was decided to limit the composition of the oil phase to four ingredients, including the oil under investigation.

All the TO SKIN and TO ACTIVE formulations reworked by the software, as well as those designed by the formulator, were prepared and analyzed in terms of active release using Franz cells.

As a final step, it was decided to further investigate the predictive capabilities of the software and its reliability by complexing the lipophilic phase. The formulator chose seven ingredients, and the software was asked to provide, by modifying the relative percentages, the TO ACTIVE and TO SKIN formulae.

### 3.1. First Phase: TO SKIN and TO ACTIVE Formulations Proposed by Formulating for Efficacy^®^ Software

In order to evaluate their formulation and predictive potential, the software was asked to propose, in qualitative (choice of excipients) and quantitative terms, a lipophilic phase optimal for the cutaneous passage of the chosen active Benzophenone-3 and one suitable for its solubilization in the formula, called TO SKIN and TO ACTIVE, respectively.

To design an O/W emulsion with a lipophilic phase equal to 24% of the total of the formulation, the inputs relative to the active (concentration equal to 5% of the total of the formulation) and to the percentage of the lipophilic phase (equal to 20.5%, excluding a concentration of 3.5% of emulsifier) were opportunely inserted, and the software was interrogated.

The software was able to identify the best two excipients, or the three best ones, for both TO SKIN and TO ACTIVE formulations.

The percentage compositions of the proposed lipophilic phases are reported in Table 3, Table 4, Table 5 and Table 6.

The formulation proposals of the software included the use of anionic surfactants (SLS and SLES) in high concentrations, especially in the TO SKIN best three ingredients formulation. These molecules are commonly used as primary surfactants in detergent products, such as shampoos, bath foams and liquid detergents. They are characterized by a high irritant power that increases with increasing concentration. The prolonged use of products containing SLES and SLS causes the drying of the skin and alteration of the hydrolipidic film, which is why they are combined with more eudermic surfactants, such as betaines or lipoprotein surfactants, which reduce skin irritation [9]. They are therefore not compatible with leave-on formulations, such as sunscreens. They would de-structure and damage the stratum corneum and promote uncontrolled transfer of the active. In addition, the BP-3 permeability predictions proposed by the software would not be reliable, as the conditions for skin integrity would not be met in vivo.

Formulating for Efficacy^®^ Software in this case also proposes the use of ingredients incompatible with the lipophilic phase; glycerin and PEG-12, included, respectively, for TO ACTIVE Best three and TO SKIN Best two, are hydrophilic ingredients and therefore inappropriate for solubilizing the active.

It should be noted that in both TO ACTIVE formulations, another sun filter, Octocrylene, was proposed as the main solvent. This is also a filter that can indeed be used as a stabilizer in combination with other sun filters (Avobenzone, for example) but not at the percentages indicated by the software prediction. The maximum content of Octocrylene allowed in Europe is 10% and, in these formulations, as they are set up, it would correspond to 19%. On the basis of these observations, it can be stated that all the lipophilic phases proposed by the software are not suitable for inclusion in a cosmetic formulation, either because of problems related to the stability of the formulation itself or in anticipation of potential use.

However, it is worth noting the difference in the composition of the two TO ACTIVE formulations compared to the two TO SKIN formulations. In the former, Octocrylene is proposed at very similar concentrations, but it is not present in the TO SKIN formulations. This leads one to think that FFE respects a logic of consistency in working in the two different modes, TO SKIN and TO ACTIVE.

In this first phase, it became evident that it was impossible to formulate the emulsions, leaving the program free to compose the lipophilic phase. It was therefore decided to change the approach in a simplified sense.

### 3.2. Second Phase: Simplified Approach

The objective of this phase was to verify the predictive reliability of the software, without investigating its formulation capabilities.

Six oils with different chemical characteristics, which are frequently used as solubilizers of sunscreens, were identified (Table 1), and six different solutions containing 5% *w/w* BP-3 were prepared.

The active ingredient was completely solubilized in all preparations (slight heating was necessary for O5), except in O1, which was therefore discarded.

### 3.3. Analysis in Franz Cells

The five oils were tested in Franz diffusion cells to analyze, under the same operating conditions, the different release of BP-3, using a synthetic cellulose membrane. For all analyses, the same raw materials and operating methods were used. The release profile of the active ingredient from the five oils was then evaluated. The experiments were repeated in quadruplicate, and the data obtained were the average of the individual values.

The cumulative amounts of the active in each cell were related to the permeation area (0.6 cm^2^) to describe the flow of the active. The data obtained in terms of µg/cm^2^ are shown below for each formulation tested (Table 7). All the results are expressed as the average of at least four different experiments plus or minus the standard deviation, which in all reposted values is less than 10%, underlining the reliability of the result.

### 3.4. Comparison with Formulating for Efficacy^®^ Software Predictions

Thanks to the BP-3 fluxes detected, it is possible to compare the release trend of the active ingredient from the different formulations analyzed. The purpose of this comparison is to understand which oils, among those tested, allow a lower release of the active ingredient and to compare them with the predictions made by Formulating for Efficacy^®^ Software, using the diffusion modeler method.

From the experimental release data (Table 7), it can be clearly seen that O5 Dicaprylyl ether behaves differently to the other oils. It has a significantly higher release capacity, which is already evident after thirty minutes, and remains higher throughout the analysis. The final amount of BP-3 released by O5 is more than double that of the other oils, which behave very similarly to each other. This result is extremely statistically significant (*p* < 0.0001 compared to all other oils, Table 8) and is consistent with the ability of the different oils to solubilize the active. In fact, the only preparation that required slight heating to achieve complete solubilization of BP-3 was O5.

These results, reported in µg/cm^2^, were expressed in percentages in order to compare them with the release predictions offered by FFE (Table 8).

To obtain the prediction data from the software, the following parameters were set: active concentration of 5%; composition of the lipophilic phase consisting of 100% of the single selected oil; membrane thickness 15 µm; quantity of formulation used 10 µm. The diffusion data obtained, defined as the diffusion output, were expressed as a percentage, similarly to the data obtained in vitro.

It was not possible to obtain a prediction of Formulating for Efficacy^®^ Software for O3, Cocoglyceride, since it is an oil composed of a heterogeneous mixture of natural glycerides, for which it is not possible to identify the exact composition percentages, and therefore, a unique Smiles code to insert it in the database.

Between the real data and those predicted by Formulating for Efficacy^®^ Software, in addition to a substantial difference of 10-1, there are clear discrepancies; what experimentally is actually the oil that releases the active ingredient in the smallest quantity (O6), for the software seems to be the oil that most favors its release. According to Software’s calculations, O4 should be the formulation that releases the least amount of BP-3. Additionally, with regard to the predictions of O5 oil, which, according to the experimental data, in addition to being the worst solvent for BP-3, is the oil showing the highest release, for the software, it is not considered as such (in fact, a higher release is predicted by O6).

In the light of these considerations, which, in general, contrast the experimental evidence with the predictions of Software, it was decided to continue the comparison process by enriching the lipophilic phase and thus moving from oils to O/W emulsions.

### 3.5. Third Phase: Verification of Software’s Formulation and Prediction Capabilities

In this phase of the research, the software was given the possibility of working with a lipophilic phase richer in components, and therefore, of modifying, in the TO SKIN or TO ACTIVE mode, the lipophilic phase of an existing emulsion designed by an expert formulator. Thus, emulsions designed by the formulator were first designed, tested and compared, and then, the software was asked to modify them.

#### 3.5.1. Emulsions Developed by an Expert Formulator

In order to design the initial emulsions (those developed by the formulator on the basis of experience), the two oils that showed the highest and lowest release capacity in the previous experimental phase were considered, i.e., those used for O5 and O6, respectively, Dicaprylyl Ether and C12–C15 Alkyl Benzoate. Therefore, a “standard” lipophilic phase consisting of four elements (including the emulsifier) and the oils contained in O5 and O6 was designed and used as the characterizing ingredients of the formulation. Two identical emulsions were then prepared, one containing O5 and the other containing O6, in the same quantities. The two emulsions were named E1 and E2, respectively (Table 9).

The objective was to investigate whether and how these oils were able to influence the release of BP-3 after inclusion in complex cosmetic formulations.

Data on BP-3 release from both formulations, which were analyzed in the Franz cell, are reported in Section 3.6.

These data confirm that, under the same conditions of analysis, the two emulsions release the active ingredient differently. The release trends obtained experimentally from the oils are confirmed. In fact, E1, which contains the same oil as O5, has a significantly higher release than E2 (which contains the same oil as O6). It can also be seen that the release of BP-3 from the emulsions is higher than that from the oils, particularly when comparing the release between O6 and E2, which is almost doubled.

#### 3.5.2. Emulsions Derived from E1 Modified by Software

To continue the study, FFE was asked to intervene in the re-modeling of the lipophilic phase of the emulsions, leaving the program free to choose which ingredients to use from those present in the emulsions designed by the formulator (E1 and E2) and in what quantities. The aims of this step were mainly threefold:

- to verify if the program is able to rework the lipophilic phase while maintaining the stability of the emulsion;
- to evaluate in Franz’s cell the effectiveness of releasing the active ingredient compared TO SKIN and TO ACTIVE;
- to compare the data obtained in vitro with the release predictions offered by the software.

In order to verify the points listed above, the E1 emulsion was taken as a reference, so neither the hydrophilic phase nor the preservative system were modified. All input parameters, relating only to the lipophilic phase, were then entered into the software. All ingredients were selected manually, excluding the emulsifier system (3.5%). The percentage of the lipophilic phase was set to 20.5%, and the active ingredient was selected, as for all formulations, at 5% *w/w*. The software was then asked to rework the composition, first in the TO SKIN mode and then in the TO ACTIVE mode, and release prediction data were collected.

Table 10 shows the two new lipophilic phases obtained, with which the E3 TO SKIN and E4 TO ACTIVE emulsions were prepared.

In the Formulating for Efficacy^®^ Software proposal, it can be seen that O5 was not considered. It was discarded in both cases. In fact, the software, already in the analysis of the single oils, did not consider it as a good ingredient to improve the release of BP-3. Furthermore, the lipophilic phase of E3 was very similar to that of E4; they differed only by 1% or 0.15% Cetearyl Alcohol.

The emulsions E3 and E4, which were subsequently produced, proved to be stable and, on initial visual and sensory inspection, very similar in consistency and skin feel to E1. They were therefore analyzed in the Franz cell, following the same procedure as for E1 and E2.

Table 11 and Section 3.6 show the results obtained experimentally and the permeability predictions offered by FFE.

In order to compare the experimental data with the predictions offered by Formulating for Efficacy^®^ Software, it was necessary, as with the oils, to report the results as a percentage.

The software was used in exactly the same way at two separate times—one to obtain the formulations and one to obtain the uptake predictions. Although the Software did not change any input parameters, when asked to replicate the rework TO SKIN from E1, it no longer produced the same formulation. As it was not possible to manually enter the percentages of each individual ingredient, the release prediction could not be obtained.

The most relevant data at this stage came from the comparison of the active releases obtained in vitro. The Software should have reworked the E3 TO SKIN and E4 TO ACTIVE formulations to increase their release, especially in the skin working mode. Instead, the results obtained show that this did not occur and that the E1 formulation developed by the formulator, in terms of the release, provided an extremely statistically significant result (*p* < 0.0001 compared to E3 and E4).

#### 3.5.3. Emulsions Derived from E2 Modified by Software

Similar to E1, for E2, the program was asked to reprocess the lipophilic phase in the TO SKIN and TO ACTIVE modes. The two lipophilic phases obtained are described in Table 12.

In contrast to what was obtained for E3 and E4, the software worked while keeping O6 in consideration for both emulsions. The differences in the composition of the two lipophilic phases are much more evident, especially with regard to the percentages used for Dibutyl Adipate and C12–C15 Alkyl Benzoate. Once again, the software confirmed its prediction of oil release; O6 was, according to Formulating for Efficacy^®^ Software, the best oil to promote the transcutaneous passage of BP-3, and in fact, it also took it into account when formulating the emulsions, unlike O5 for E3 and E4.

At this point, the E5 and E6 emulsions, prepared with the so called “English method”, in which the emulsifier is solubilized/dispersed in the phase in which it is more soluble, were stable and, despite the major differences in composition, very similar to each other in terms of consistency and skin feel.

Similar to the other formulations, E5 and E6 were analyzed in Franz diffusion cells. The experimentally obtained data and the software predictions are reported in Table 13 and Table 14.

This analysis offers a very important finding. The E5 TO ACTIVE emulsion, which contains a high percentage of O6 (C12–C15 Alkyl Benzoate), releases the active more than E6 TO SKIN. This highlights two critical points. The software should formulate an optimal lipophilic phase in the TO SKIN mode, but it does so for TO ACTIVE, and it also does not do so consciously, given the predictive release data shown in the table below (Table 13).

As can be seen, the release predicted by Formulating for Efficacy^®^ Software for E6 is lower than that predicted for E5. The software, therefore, characterizes, as always, the E5 TO SKIN formulation as the best performing, but the experimental data confirm that the formulation that most favors release, even compared to E2 (the one prepared by the formulator), is E6, with an extremely statistically significant result (*p* <0.0001).

### 3.6. Comparison of Data Obtained from Emulsion Analysis

In the following table (Table 14), all the data collected from the Franz diffusion cells are reported to show more clearly the trend of BP-3 release from the different emulsions.

All the results are expressed as the average of at least four different experiments plus or minus the standard deviation, which in all reposted values is less than 10%, underlining the reliability of the result.

**Table 14:** Cumulative BP-3 release from all prepared formulations. Q is the quantity of compound traversing the membrane in time t, and A is the area of exposed membrane in cm^2^. Each value was obtained from four experiments (mean ± SE).

	Formulator O5	To Skin O5	To Active O5	Formulator O6	To Skin O6	To Active O6
Time (minutes)	BP3 from E1 Q/A (µg/cm^2^)	BP3 from E3 Q/A (µg/cm^2^)	BP3 from E4 Q/A (µg/cm^2^)	BP3 from E2 Q/A (µg/cm^2^)	BP3 from E5 Q/A (µg/cm^2^)	BP3 from E6 Q/A (µg/cm^2^)
0	0	0	0	0	0	0
30	42.22 ± 3.62	27.45 ± 0.27	27.51 ± 1.97	47.93 ± 0.43	37.16 ± 0.33	35.03 ± 0.41
60	98.27 ± 1.26	71.39 ± 0.76	65.44 ± 5.05	69.11 ± 1.95	59.97 ± 0.74	72.20 ± 1.37
120	188.65 ± 7.34	130.52 ± 4.85	133.79 ± 8.48	138.24 ± 1.45	113.42 ± 1.5	144.73 ± 5.90
180	257.71 ± 2.81	139.26 ± 1.48	151.76 ± 2.90	175.32 ± 4.14	167.11 ± 1.9	187.03 ± 2.31
240	308.55 ± 3.50	167.00 ± 1.67	192.67 ± 1.17	193.08 ± 4.56	199.88 ± 1.7	217.71 ± 2.96
300	334.09 ± 3.11	184.50 ± 3.88	208.73 ± 3.94	221.33 ± 4.71	226.33 ± 3.5	240.82 ± 4.15
360	344.61 ± 5.15	215.83 ± 3.83	209.39 ± 2.96	232.04 ± 5.50	223.61 ± 2.9	250.26 ± 5.24

It can be seen that the E1 formulation (designed by the formulator and containing O5) is the one that releases the most active ingredient throughout the analysis. This behavior is contrary to Software’s predictions. O5 was not considered a good solvent for BP-3 release already in the single oil analysis, so it was discarded by the program in the design of the two emulsions, E3 TO SKIN and E4 TO ACTIVE, which should have been better performing than E1 but did not turn out to be so. On the contrary, they actually showed the worst performance of all the emulsions tested.

From this first consideration, it is possible to understand how the software made a basic error in the evaluation of the ingredient Dicaprylyl Ether (O5), which was also propagated in the subsequent formulations proposed by the software. The TO SKIN formula, according to the designers of the software, should always be an improved proposal, in terms of the release, of the starting formula, and in this case, it was not. The release predictions offered by Formulating for Efficacy^®^ Software were also discordant with the data obtained in vitro (Table 14), but the difference between TO SKIN and TO ACTIVE was reconfirmed. In fact, the relationship between the two formulations, which predicted a greater release for TO SKIN than TO ACTIVE, was always maintained. These results are similar to some already obtained by us in the past (unpublished data) [3].

Comparing the release of BP-3 from E2 with E5 and E6, other discrepancies can be observed between the behavior of the emulsions predicted by Formulating for Efficacy^®^ Software and the real one. The use of C12–C15 Alkyl Benzoate (O6) was confirmed to be effective in improving the permeability of the active (however, less than O5). In fact, E2 proposed by the formulator and E6 TO ACTIVE were among the most efficient. However, the program did not detect this correlation, since it used higher concentrations of O6 to formulate not the proposed TO SKIN improvement but the TO ACTIVE one; it therefore recognized a modulation capacity of the release in O6 but did not exploit it for the working mode that should increase the passage of the active more than any other, i.e., the TO SKIN one. In this phase, the main principle of the FFE working modes regarding the relationship between TO SKIN and TO ACTIVE was not respected. E6 TO ACTIVE guaranteed a greater release compared to E5 TO SKIN and also compared to all the other emulsions analyzed, except for E1, which remained the best performing. The release predictions provided by the software and shown in Table 11 and Table 13 were thus subverted.

## 4. Discussion

In this study, thanks to the preliminary simplified approach, it was possible to verify how the software classified, according to the release prediction data, the different oils selected by the formulator in terms of the ability to deliver BP-3 in the skin. The software’s considerations regarding the best and worst oil to release the active ingredient were also confirmed in the next experimental phase, i.e., for the formulation of emulsions. C12–C15 Alkyl Benzoate (O6) was considered by Formulating for Efficacy^®^ Software to be the best performing. In fact, it was also maintained in the composition of the emulsions, unlike Dicaprylyl Ether (O5). However, the experimental results obtained from the Franz cells contradicted the predictions of the software, both for oils and emulsions. The analysis of the emulsions showed that Formulating for Efficacy^®^ Software did not improve the release capacity of the active ingredient, especially for the two TO SKIN formulations derived from E1 and E2.

In the data analysis, it was also highlighted that the E6 TO ACTIVE emulsion showed a higher active release than E5 TO SKIN, disregarding the expectations based on previous approaches to the software.

According to the results reported in the analysis of the oils, it would have been advisable to use C12–C15 Alkyl Benzoate to formulate the emulsions, as it is the oil that least promotes release. However, Formulating for Efficacy^®^ Software, by formulating E5, and especially E6, demonstrated that it is possible to increase BP-3 release by using it. This was particularly relevant, as C12–C15 Alkyl Benzoate is commonly reported, and therefore used, as a good solubilizer for sunscreens [18].

Poor compatibility between BP-3 and Dicaprylyl Ether was also observed during the preparation of the O5 solution (the only one that required heating). This showed that the low affinity between the two reflexively increased the release of BP-3, both from the single oil (O5) and the emulsion (E1), so the use of this ingredient is not advisable.

By also examining the emulsions derived from E1, the Software showed that the small compositional variation between E3 and E4, consisting of 0.15% Cetearyl Alcohol, can be reflected with a minimal difference in release. This indicates to the formulator that the ingredient in question should indeed be used in moderation or possibly avoided.

In recent years, only a few authors have attempted the application of HSP to make the formulator’s work more predictable, also through the use of expert software, such as the Formulating for Efficacy^®^ Software that was created using the HSPiP principles. Lane et al. [19] reported some interesting works, in particular that of Oxybutin (O) permeation in the skin at finite doses in relation to the solvent used. The study demonstrated that the amount of O that reached the stratum corneum was greater for octyl salicylate (OS) than for propylene glycol (PG) with a linear relationship for OS, in line with the higher solubility in OS of O. However, when considering the amount of O that penetrated the skin, they found a completely different picture. This was because the greater permanence in the skin of OS, which is more liposoluble than PG, consented a better solubility of O. On the other hand, the PG penetrated through the skin more quickly (8 h vs. 24 h); thus, O started to crystalize and was no longer able to permeate through the skin, and up to 80% was not penetrated. They also reported in another example [19] on Ibuprofen that under the “infinite” dose condition, the major driving force is the partition coefficient. However, when the amounts are significantly different under the “finite” dose condition, the solubility is the major determinant rather than the partition coefficient. As explained by Abbott [4], with the same approach, Michniak-Kohn et al. investigated the effects of penetration enhancers, also demonstrating that their efficacy depends on the solubility parameters, physicochemical interaction and thermodynamic activity [20]. The Formulating for Efficacy^®^ Software was chosen because of the integrated calculation of the ingredient active gap (IAG)/ingredient skin gap (ISG) solubility for the active in the formulation (SolV) and formulation solubility in the skin (SolvS). Thus, the Hansen solubility parameters of the actives and excipients in combination with Software were used to describe the variation of drugs crossing the biological membrane in relation to enhancers/excipients, using nicotine as the model active molecule. They demonstrated how, understanding the properties and solubility of the active and the enhancers, the interaction of the latter with the active and the skin simplified the prediction and comprehension of the enhancers’ behavior. Furthermore, as anticipated above, non-steroidal anti-inflammatory drugs (NSAIDs) are a very interesting field of topical applications but always with problems related to their lipophilic nature and low skin permeability. Jameel et al. [21] were also intrigued by the unique feature of HSPiP and Formulating for Efficacy^®^ Software that they applied in the design of emulgels for the topical application of Iburpofen. In a 360-degree approach, they designed and optimized the emulgels using the Software, next investigating the physicochemical properties, spreadability, appearance, droplet size, stability and performance (i.e., in vitro drug release and permeation and in vivo permeation). During this study, 163 ingredients were evaluated for their potential penetration booster capability and, on the basis of the diffusion modeler suggestions, only 5 were selected for testing with a consistent saving of time. As a result of the study, very different penetration enhancers (lipophilic) combined with Dimethyl Isosorbide (DMI, hydrophilic) were the best combination for Ibuprofen penetration into the skin, thus demonstrating that Formulating for Efficacy^®^ Software worked well in calculating the optimal composition of one phase (water or oil at a time) for a given active at a given concentration to reach maximum solubility and high activity. Encouraged by these results, we decided to challenge the software with a complex topic, such as that of sunscreen design, due to the growing concerns over possible induced dermatitis, which would require a good spreading on the skin, and thus, good dissolution in the formulation but a reduced penetration through the skin. To this aim, we investigated the correlation between the composition of a lipophilic phase and the release capacity of Benzophenone-3 using the Software as a support for the development phase of new sunscreen formulations.

From the various tests carried out in the study phase of the software and the data collected in the experimental phase, it was possible to identify the software’s “strong points”, which characterize its usefulness in certain situations, as shown above, as well as its limitations of use:

(1) Advantage: The three-dimensional representation of the different substances (active ingredient, skin, ingredients and formulation) in Hansen’s space is an effective visual and immediate method for obtaining a preliminary assessment of the overall behavior of the formulation [9]. Although it is not an immediately comprehensible model, it allows the operator, even with minimal experience, to understand the mechanisms and logic behind Hansen’s theory. Disadvantage: The criticality of this model could be identified in the lack of possibility to represent the already existing lipophilic phases. As highlighted in the study for E1 and E2, the lipophilic phases proposed by the formulator cannot be inserted as such in the software. The possibility given by the software is to be able to select the individual ingredients but not the actual quantities of each, so it is impossible to have a graphical representation and, consequently, the permeability prediction data for these formulations.

(2) Disadvantage: The software did not perform well in the autonomous formulation of lipophilic phases. In fact, in the first phase of the study, Formulating for Efficacy^®^ Software, left free in the selection of the ingredients, proposed inadequate and probably unstable formulations, which were therefore neither produced nor analyzed in Franz’s cell. Advantage: However, on the basis of the indications provided by an expert formulator, FFE was able to produce lipophilic phases, which, when inserted into the emulsion, were stable and sometimes even more pleasant in appearance and consistency than the emulsions taken as reference, i.e., E1 and E2, prepared by the formulator.

(3) Disadvantage: A limitation of the Software, which should possibly be resolved, is the inability to exploit the potential of raw materials of natural origin. The possibility of inserting such mixtures is not foreseen due to their variability and heterogeneity, which make them incompatible with the system of inserting ingredients using the SMILES codes [14]. Working on cosmetic formulations without being able to consider indispensable raw materials, such as those of natural origin, is tantamount to depriving the formulations produced of numerous properties that characterize these substances. This limitation thus distances the software from the actual intended use of cosmetic products. It would also be wrong to use such natural raw materials and not to consider them in the forecasts for the release of the actives, as they would, to all intents and purposes, constitute the lipophilic phase, varying its chemical composition and thus influencing its activity for the release of the actives.

## 5. Conclusions

In conclusion, it is possible to state that, in our case, there was no agreement between the experimental data collected and Software’s predictions and that the Software did not overall improve the prediction ability of the active to cross the stratum corneum.

However, considering that the aim of the study was not to increase BP-3 absorption but rather to decrease it, it was possible to understand which of the most commonly used ingredients in sunscreens could limit its release from the formulation without resorting to complex formulation technologies, such as microencapsulation [22] or the use of nanostructured lipid carriers [1].

Given all the considerations put forward, it is possible to affirm that, despite the criticalities that emerged, the use of the Formulating for Efficacy^®^ Software can be exploited to modulate the release of the active ingredients from articulated formulations, such as cosmetic emulsions. However, in its use, the intervention of an operator expert in formulation was, in our case, indispensable, in order to discriminate the choices of the software, which were not always reliable. The figure of the formulator is also indispensable in order to structure the finished product, since the program does not deal with the formulation of the hydrophilic phase, the choice of emulsifier and the preservative system.

Indeed, in other, simpler cases described in the literature related to penetration enhancers and to design and optimization of computer-aided formulations to improve the delivery of ibuprofen through the skin [20,21], the software performed well, and the question still remains open because with proper input from an experienced formulator, the software can produce better outcomes than those from an expert formulator alone. As a matter of fact, the real issue may not be that the Formulating for Efficacy^®^ Software does not work in complex cases, but since it cannot determine the final objectives of the formulator, it may end up producing useless information in the end. Further studies, currently ongoing, are trying to highlight what would be the improvement in time to development of the use of Formulating for Efficacy^®^ Software by non-experienced formulators.

## Figures and Tables

**Table 1:** List of selected oils.

Formulation	Solvent
O1	Paraffinum Liquidum
O2	Dicaprylyl Carbonate
O3	Cocogliceride
O4	Caprilic-Capric Triglyceride
O5	Dicaprylyl Ether
O6	C12–C15 Alkyl Benzoate

**Table 2:** List of ingredients of the formulations studied.

	E1	E2	E3	E4	E5	E6
Aqua	-	-	-	-	-	-
Glycerin	-	-	-	-	-	-
Disodium EDTA	-	-	-	-	-	-
Carbomer	-	-	-	-	-	-
Tribehenin PEG-20 esters	-	-	-	-	-	-
Squalene	-	-	NP	NP	NP	NP
Dibutyl Adipate	-	-	-	-	-	-
Cetearyl Alcohol	-	-	-		-	-
Dicaprylyl ether	-	NP	NP	NP	NP	NP
C12.C15 Alkyl benzoate	NP	-	NP	NP	-	-
Benzophenone-3	-	-	-	-	-	-
1,2-Hexanediol, 1,2-Octanediol and Tropolone	-	-	-	-	-	-

NP means not present in the formula.

**Table 3:** Formula TO SKIN—Best two ingredients.

Ingredients	Percentage
PEG-12	86%
SLES (Sodium Laureth Sulfate)	14%

**Table 4:** Formula TO ACTIVE—Best two ingredients.

Ingredients	Percentage
Octocrylene	79%
SLS (Sodium Lauryl Sulfate)	21%

**Table 5:** Formula TO SKIN—Best three ingredients.

Ingredients	Percentage
Hydrogenated polyisobutene (MW268)	60%
SLS	35%
SLES	5%

**Table 6:** Formula TO ACTIVE—Best three ingredients.

Ingredients	Percentage
Octocrylene	80%
SLS	14%
Glycerine	6%

**Table 7:** Cumulative active release from the different oils: Q is the quantity of compound traversing the membrane in time t, and A is the area of exposed membrane in cm^2^. Each value was obtained from four experiments (mean ± SE).

Time (min)	BP3 from O2 Q/A (µg/cm^2^)	BP3 from O3 Q/A (µg/cm^2^)	BP3 from O4 Q/A (µg/cm^2^)	BP3 from O5 Q/A (µg/cm^2^)	BP3 from O6 Q/A (µg/cm)
0	0	0	0	0	0
30	22.80 ± 0.42	28.58 ± 0.52	17.46 ± 0.29	59.75 ± 2.22	2.16 ± 0.14
60	32.33 ± 0.51	48.72 ± 1.30	31.68 ± 0.35	78.89 ± 1.27	18.96 ± 0.19
120	63.26 ± 0.63	79.82 ± 0.91	64.43 ± 0.63	140.19 ± 1.90	56.83 ± 3.70
180	88.76 ± 1.07	90.95 ± 1.55	84.35 ± 0.21	174.53 ± 4.38	70.76 ± 1.05
240	110.29 ± 2.60	111.11 ± 2.30	107.83 ± 1.29	220.48 ± 4.91	94.52 ± 1.09
300	133.75 ± 1.14	124.47 ± 1.60	124.41 ± 1.55	250.78 ± 0.50	106.76 ± 7.01
360	129.91 ± 8.06	138.76 ± 2.46	138.83 ± 3.67	291.65 ± 3.14	121.57 ± 1.48

O2 (Dicaprylyl Carbonate); O3 (Cocogliceride); O4 (Caprilic-Capric Triglyceride); O5 (Dicaprylyl Ether); O6 (C12-C15 Alkyl Benzoate)

**Table 8:** Release rates obtained from Franz cells and predicted by the software by the different oil formulations: O2 (Dicaprylyl Carbonate); O3 (Cocogliceride); O4 (Caprilic-Capric Triglyceride); O5 (Dicaprylyl Ether); O6 (C12-C15 Alkyl Benzoate). Each value was obtained from four experiments (mean ± SE). *** *p* < 0.0001.

Formulation	% of BP-3 Released from 1 g Formula of 5%	% of BP-3 Released Prediction
O2	0.156 ± 0.008 ***	0.0243
O3	0.167 ± 0.003 ***	- *
O4	0.167 ± 0.004 ***	0.0149
O5	0.350 ± 0.003 ***	0.0184
O6	0.146 ± 0.001 ***	0.0273

* Data not obtained.

**Table 9:** Percentage composition of emulsion E1, containing O5 (Dicaprylyl ether), and emulsion E2, containing O6 (C12–C15 Alkyl benzoate).

	Emulsion E1	Emulsion E2
**PHASE 1**	**75.5%**	**75.5%**
Aqua	72.25%	72.25%
Glycerin	3.0%	3.0%
Disodium EDTA	0.1%	0.1%
Carbomer	0.15%	0.15%
**PHASE 2**	**24%**	**24%**
Tribehenin PEG-20 esters	3.5%	3.5%
Squalene	3.0%	3.0%
Dibutyl Adipate	4.0%	4.0%
Cetearyl Alcohol	1.5%	1.5%
**Dicaprylyl ether**	**7.0%**	**-**
**C12-C15 Alkyl benzoate**	**-**	**7.0%**
Benzophenone-3	5.0%	5.0%
**PHASE 3**	**0.5%**	**0.5%**
1,2-Hexanediol, 1,2-Octanediol e Tropolone	0.5%	0.5%
**TOTAL (ALL PHASES)**	**100%**	**100%**

**Table 10:** Lipophilic phase of E3 TO SKIN emulsion and E4 TO ACTIVE emulsion.

	E3 TO SKIN	E4 TO ACTIVE
PHASE 2	24%	24%
Tribehenin PEG-20 Esters	3.5%	3.5%
Dibutyl Adipate	15.35%	15.5%
Cetearyl Alcohol	0.15%	-
Benzophenone-3	5.0%	5.0%

**Table 11:** Percentage release of BP-3 from E1, E3 and E4. Each value was obtained from four experiments (mean ± SE). *** *p* < 0.0001.

Formulation	% of BP-3 Released from 1 g Formula of 5%	% of BP-3 Released FFE Prediction
E1 with O5	0.385 ± 0.005 ***	-
E3 TO SKIN (without O5)	0.241 ± 0.004 ***	- *
E4 TO ACTIVE (without O5)	0.234 ± 0.003 ***	0.273

* The software did not predict the formula that was analyzed.

**Table 12:** Lipophilic phase of E5 TO SKIN emulsion and E6 TO ACTIVE emulsion.

	E5 TO SKIN	E6 TO ACTIVE
PHASE 2	24%	24%
Tribehenin PEG-20 Esters	3.5%	3.5%
Dibutyl Adipate	15.2%	5.27%
Cetearyl Alcohol	0.15%	0.15%
C12-C15 Alkyl Benzoate	0.15%	10.08%
Benzophenone-3	5.0%	5.0%

**Table 13:** Percentage release of BP-3 from emulsions E2, E5 and E6 containing O6 (C12-C15 Alkyl Benzoate). Each value was obtained from four experiments (mean ± SE). ** *p* < 0.005, *** *p* < 0.0001.

Formulation	% of BP-3 Released from 1 g Formula of 5%	% of BP-3 Released Prediction
Emulsion 2 with O6	0.259 ± 0.005 ***	-
E5 TO SKIN with O6	0.250 ± 0.003 **	0.273
E6 TO ACTIVE with O6	0.279 ± 0.005 ***	0.236

## References

[1] Gilbert E., Roussel L., Serre C., Sandouk R., Salmon D., Kirilov P., Haftek M., Falson F., Pirot F. (2016). Percutaneous absorption of benzophenone-3 loaded lipid nanoparticles and polymeric nanocapsules: A comparative study. Int. J. Pharm..

[2] Ragno A., Baldisserotto A., Antonini L., Sabatino M., Sapienza F., Baldini E., Buzzi R., Vertuani S., Manfredini S. (2021). Machine learning data augmentation as a tool to enhance quantitative composition–activity relationships of complex mixtures. A new application to dissect the role of main chemical components in bioactive essential oils. Molecules.

[3] Bino A., Baldisserotto A., Scalambra E., Dissette V., Vedaldi D.E., Salvador A., Durini E., Manfredini S., Vertuani S. (2017). Design, synthesis and biological evaluation of novel hydroxy-phenyl-1H-benzimidazoles as radical scavengers and UV-protective agents. J. Enzyme Inhib. Med. Chem..

[4] Abbott S. (2012). An integrated approach to optimizing skin delivery of cosmetic and pharmaceutical actives. Int. J. Cosmet. Sci..

[5] Wiechers J.W., Kelly C.L., Blease T.G., Dederen J.C. (2004). Formulating for efficacy. Int. J. Cosmet. Sci..

[6] Abbott S. (2022). https://www.hansen-solubility.com/.

[7] Aulton M.E., Taylor K.M.G. (2013). Aulton’s Pharmaceutics: The Design and Manufacture of Medicines.

[8] Van der Westhuizen J. (2014). Formulation, In Vitro Release and Transdermal Diffusion of Atropine by Implementation of the Delivery Gap Principle. Master’s Thesis.

[9] JW Solutions Software, FFE Help and Tutorial, Official Site. https://www.jwsolutionssoftware.com.

[10] Hansen Solubility Parameters, Official Site. https://www.hansen-solubility.com/HSP-science/for-beginners.php.

[11] Li C.C., Li Y.T., Chen Y.T., Sie S.F., Chen-Yang Y.W. (2015). Improvement in UV protection retention capability and reduction in skin penetration of Benzophenone-3 with mesoporous silica as drug carrier by encapsulation. J. Photochem. Photobiol. B.

[12] Hsu H.H., Kracht J.K., Harder L.E., Rudnik K., Lindner G., Schimek K., Marx U., Pörtner R. (2018). A Method for Determination and Simulation of Permeability and Diffusion in a 3D Tissue Model in a Membrane Insert System for Multi-well Plates. J. Vis. Exp..

[13] Louwerse M.J., Maldonado A., Rousseau S., Moreau-Masselon C., Roux B., Rothenberg G. (2017). Revisiting Hansen solubility parameters by including thermodynamics. ChemPhysChem.

[14] Tampucci S., Burgalassi S., Chetoni P., Monti D. (2018). Cutaneous Permeation and Penetration of Sunscreens: Formulation Strategies and In Vitro Methods. Cosmetics.

[15] Li C.C., Lin L.H., Lee H.T., Tsai J.R. (2016). Avobenzone encapsulated in modified dextrin for improved UV protection and reduced skin penetration. Chem. Pap..

[16] Martins R.M., Siqueira S., Fonseca M.J., Freitas L.A. (2014). Skin penetration and photoprotection of topical formulations containing benzophenone-3 solid lipid microparticles prepared by the solvent-free spray-congealing technique. J. Microencapsul..

[17] DiNardo J.C., Downs C.A. (2018). Dermatological and environmental toxicological impact of the sunscreen ingredient oxybenzone/benzophenone-3. J. Cosmet. Dermatol..

[18] Sohn M., Amorós-Galicia L., Krus S., Martin K., Herzog B. (2020). Effect of emollients on UV filter absorbance and sunscreen efficiency. J. Photochem. Photobiol. B Biol..

[19] Lane M., Hadgraft J., Oliveira G., Vieira R., Mohammed D., Hirata K. (2012). Rational formulation design. Int. J. Cosmet. Sci..

[20] Haq A., Chandler M., Michniak-Kohn B.B. (2020). Solubility-physicochemical-thermodynamic theory of penetration enhancer mechanism of action. Int. J. Pharm..

[21] Jameel B.M., Huynh A., Chadha A., Pandey S., Duncan J., Chandler M., Baki G. (2019). Computer-based formulation design and optimization using Hansen solubility parameters to enhance the delivery of ibuprofen through the skin. Int. J. Pharm..

[22] Klimaszewska E., Wieczorek D., Zięba M., Małysa A., Staszak K., Kwaśniewska D., Adamczyk K., Drzymala K., Dobrowolski A. (2018). Effect of N-dodecyl-N-(propylpiperydinium-3-sulfonate) on usage properties of liquid soaps for sensitive skin. Tenside Surfact. Det..

